# Oligoclonal Band Status and Features of Radiological and Clinical Findings in Patients with Multiple Sclerosis in Lithuania

**DOI:** 10.3390/medicina59061028

**Published:** 2023-05-26

**Authors:** Emilija Aleksandravičiūtė, Radvilė Stankevičiūtė, Renata Balnytė, Laurynas Šaknys, Ingrida Ulozienė

**Affiliations:** 1 Department of Neurology, Lithuanian University of Health Sciences Medical Academy, A. Mickevičiaus g.9, LT-44307 Kaunas, Lithuania; 2 Department of Otorhinolaringology, Lithuanian University of Health Sciences Medical Academy, A. Mickevičiaus g.9, LT-44307 Kaunas, Lithuania

**Keywords:** multiple sclerosis, magnetic resonance imaging, oligoclonal bands, expanded disability status scale

## Abstract

*Background and Objectives*: Multiple sclerosis (MS) is a widely spread and debilitating disease with 2.8 million people worldwide currently affected. However, the exact pathogenesis of the disease and its progression remains incompletely understood. According to the revised McDonald criteria, cerebrospinal fluid oligoclonal bands (CSF OCBs) magnetic resonance imaging (MRI) results, in conjunction with clinical presentation, remain the gold standard of MS diagnostics. Therefore, this study aims to evaluate the association between CSF OCB status and features of radiological and clinical findings in patients with multiple sclerosis in Lithuania. *Materials and Methods*: The selection of 200 MS patients was performed in order to find associations between CSF OCB status, MRI data and various disease features. The data were acquired from outpatient records and a retrospective analysis was performed. *Results*: OCB positive patients were diagnosed with MS earlier and had spinal cord lesions more frequently than OCB negative patients. Patients with lesions in the corpus callosum had a greater increase in the Expanded Disability Status Scale (EDSS) score between their first and last visit. Patients with brainstem lesions had higher EDSS scores during their first and last visit. Even so, the progression of the EDSS score was not greater. The time between the first symptoms and diagnosis was shorter for patients who had juxtacortical lesions than patients who did not. *Conclusions*: CSF OCBs and MRI data remain irreplaceable tools when diagnosing multiple sclerosis as well as prognosing the development of the disease and disability.

## 1. Introduction

Multiple sclerosis (MS) is a widely spread and debilitating disease with 2.8 million people worldwide currently affected [1]. Even in a relatively short period of time of 2 years, at least one third of MS patients report disability progression [2]; however, the exact pathogenesis of the disease and its progression remains incompletely understood [3].

Cerebrospinal fluid oligoclonal bands (CSF OCBs) can also be determined as clonal immunoglobulin G (IgG) antibodies produced by B and plasma cells locally in the central nervous system (CNS) [4] and can be found in up to 85% of MS patients [3]. Nowadays, they play a key role in the diagnostics of MS while presenting a prognostic value as well. According to the revised 2017 McDonald Criteria for the Diagnosis of Multiple Sclerosis, positive CSF OCBs can substitute for dissemination in time, leading to a faster diagnosis and treatment [5].

When predicting the progression of CIS (clinically isolated syndrome) to MS, CSF OCB positive patients are found to be more likely to develop the disease compared to CSF OCB negative patients [6,7]. Moreover, a meta-analysis demonstrated that OCB positive MS patients are more susceptible to the development of disability, and a more severe disease can usually be present [7]. A small study has also shown that the presence of CSF OCBs could determine disease activity and their presence could be modulated by certain medications [8]; however, the topic needs further investigation.

On the other hand, CSF OCB is not a definite measurement; therefore, obtaining the MRI (magnetic resonance imaging) is mandatory when MS is suspected. Up to this day, combined with clinical presentation, it remains the gold standard in the diagnostics of MS [9]. Therefore, it has a high predictive value for distinguishing early multiple sclerosis and allows monitoring of the disease as well [10].

Studies have shown that the reduction in MRI lesions results in disease remission and could be used as a monitoring tool at the beginning and later stages of the disease [11]. Moreover, MRI data, such as chronic white matter lesion activity, have been proven to predict disease progression [12]. T2 lesion volume has been associated with poor clinical outcomes as well [13]. However, some studies suggest ambivalent results when trying to associate changes in MRI (such as brain volume loss) with the disability progression in MS patients, meaning that further investigation is needed [14,15]. A shortage of data on how different lesion localizations affect disability is faced. However, lesions in the periventricular white matter and left internal capsule were demonstrated to cause the most severe drop in functional outcomes [16]. Thus, it could be hypothesized that different MRI features would lead to various clinical outcomes as well.

Therefore, this study aims to evaluate the association between CSF OCB status and features of radiological and clinical findings in patients with multiple sclerosis in Lithuania.

## 2. Materials and Methods

The selection of 200 MS patients treated in the LUHS (Lithuanian University of Health Sciences) Kaunas Clinics from 1 January 2000 to 31 December 2020 was performed in order to find associations between CSF OCB status, MRI data and various disease features. Approximately 1200 MS patients are treated in the LUHS. The estimated percentage of positive CSF OCBs in MS patients is about 85% [7]. Therefore, the recommended sample size is 169 patients (z = 95%, E = 5%). The data were acquired from outpatient records, and a retrospective analysis was performed. Variables included in the selection were patient age (at diagnosis and first symptoms), sex, course of the disease, EDSS (Expanded Disability Status Scale) scores at the first (EDSS1) and the last (EDSS2) visit, MRI data and CSF OCB positivity status. Additionally, the difference between EDSS2 and EDSS1 was calculated, allowing the assessment of the progression of disability. Furthermore, in cases where at least 2 MRI scan results were available, the results were compared, and the appearance of new lesions and overall lesion activity was evaluated. Changes in MRI were grouped to negative (increased lesion activity/number) and stable/positive (decreased or stable lesion activity).

The study included patients with confirmed MS diagnosis only. MS diagnosis was established according to widely accepted and revised McDonald criteria. Lumbar puncture and CSF examination were performed at the time of the diagnosis. CSF samples were analyzed using isoelectric focusing and IgG specific immunofixation with the purpose of testing for the presence of intrathecal specific IgG OCBs and comparing directly with the serum samples. Positive OCBs were defined when more than 2 bands were present in the CSF, while absent in the corresponding blood serum. Demographic, clinical data and results of magnetic resonance imaging were collected for all patients. Disability was measured using the Kurtzke Expanded Disability Status Scale.

Statistical analysis was performed using the SPSS (Statistical Package for the Social Sciences) tool (IBM Corp. Released 2020. IBM SPSS Statistics for Windows, Version 27.0. Armonk, NY: IBM Corp) Qualitative variables were analyzed using the chi-square test, whereas quantitative variables were analyzed using the independent samples *t* test and the Mann–Whitney test. Results were interpreted as statistically significant when *p* < 0.05.

Ethical approval was obtained from the local LUHS Department of Bioethics (Approval No. BEC-MF-50).

## 3. Results

Out of the 200 reviewed MS cases, 65 (32.5%) were male patients and 135 (67.5%) were female. The mean age at the time of the diagnosis was 36.4 ± 11.668, ranging between 18 and 74 years. The mean age at the time of the first symptoms was 32.13 ± 10.790 and ranged from 12 to 73 years. The mean time from the first symptoms to the diagnosis was 5.07 ± 9.35, ranging from 0 (diagnosed in the same year symptoms manifested) to 56 years. A total of 95% (195) of the patients had relapsing–remitting multiple sclerosis (RR). Nine patients had secondary progressive (SP) multiple sclerosis, and one patient had primary progressive multiple sclerosis (PP). The mean EDSS1 score was 2.28 ± 1.16, ranging from 0 to 7, and the mean EDSS2 score was 3.17 ± 1.77, ranging between 0 and 9.5. The mean difference between EDSS2 and EDSS1 (EDSS2–EDSS1) was 0.86 ± 1.66. The value ranged from −4.5 and 6.5 (negative values show improvement of disability, while positive values show worsening).

A total of 184 (92%) of the cases had at least one lesion in the periventricular area. It was the most common localization of MS lesions in our study. Additionally, almost three quarters (148 (74%)) of the patients had lesions in the corpus callosum. Juxtacortical lesions were found in 131 (66.2%) of the cases. A total of 80 patients (56.7%) had spinal cord lesions. Half of the patients (100 (50%)) had brainstem lesions. Less than half of the patients (94 (47%)) had cerebellar lesions, meaning that cerebellar lesion localization was the least common in our study.

Most cases had data of at least two MRI scans, allowing the assessment of new lesion appearance and changes of lesion activity. A total of 74 (37.4%) cases had negative changes, and 124 (62.6%) cases had positive changes or stable results in MRI (Table 1).

### 3.1. CSF OCBs and Their Relation to Disease Characteristics

**Figure 1:**
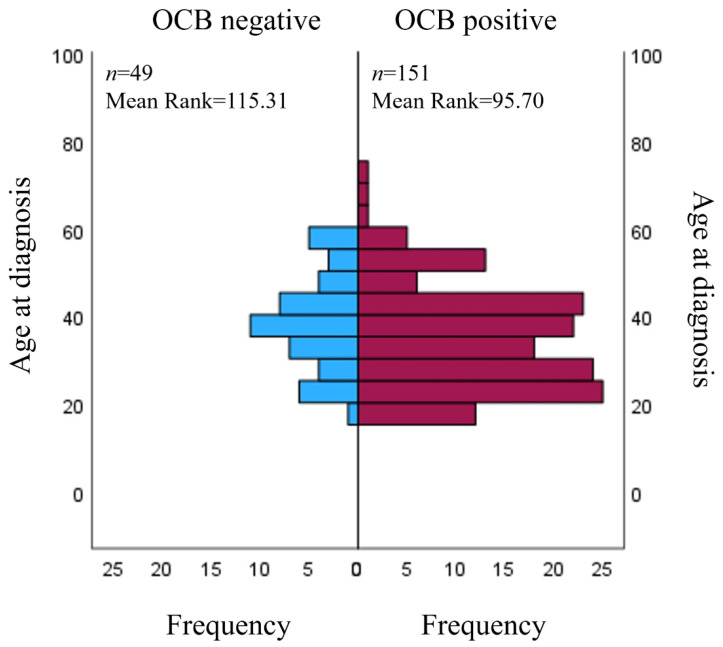
Relation between OCB status and age at diagnosis. OCB: oligoclonal band.

Positive CSF OCBs were found in 151 (75.5%) of the cases; 49 (24.5%) of them were negative. There were no statistically significant associations between OCB positivity status and sex. Furthermore, OCB positive patients (median = 35 (18–74)) were diagnosed with MS earlier than OCB negative patients (median = 39 (20–59), U = 3084, *p* = 0.039) (Figure 1). There were no relations between the OCB positivity results and patient age at the symptom onset, EDSS scores of the first and last visit and its change through time.

A total of 141 patients had MRI scans of the cervical spinal cord; 80 of them (56.7%) had spinal cord lesions. The majority of patients (81.3%) with spinal cord lesions were OCB positive, meaning that spinal cord lesions were more frequently found in OCB positive patients (χ^2^ = 4.473, *p* = 0.034). Other MRI lesion localizations (periventricular, c. callosum, brainstem, cerebellar, juxtacortical), as well as the progression and number of MRI lesions, were not associated with OCB positivity status (Table 2).

### 3.2. Features of Radiological Findings and Their Relation to Disease Characteristics

The vast majority (198 (99%)) of the cases had the data of at least two MRI scans, allowing the evaluation of the appearance of new lesions and disease progression.

Periventricular, cerebellar and spinal cord lesions did not have a relation to the patient age at the time of the first symptoms and diagnosis as well as the EDSS score.

The lesions of the corpus callosum were not associated with the patient age at the time of the first symptoms and diagnosis. However, patients who had lesions in the corpus callosum (median = 1 (−3–6.5)) had a greater increase in the EDSS score between their first and last visit (U = 4560.5, *p* = 0.045) compared to patients with no callosal lesions (median = 0.5 (−4.5–6.5)).

No relation was observed between the brainstem lesions and the patient age at the time of the first symptoms and diagnosis. Patients with brainstem lesions had higher EDSS scores during their first visit (median = 2.5 (0–6.5) than patients without brainstem lesions (median = 2 (0–7) (U = 6281.5, *p* = 0.002). Moreover, higher EDSS scores during the last visit were observed in patients with brainstem lesions (median = 3.5 (1–7.5) compared to patients with no brainstem lesions (median = 2.5 (0–9.5) (U = 6041, *p* = 0.004). Although, the difference of the EDSS scores between the first and last visit was not any different between the patients with and without brainstem lesions.

The juxtacortical lesions did not have a relation to the patient age at the time of the first symptoms and diagnosis, or to the EDSS score. The time between the first symptoms and the diagnosis was shorter for the patients who had juxtacortical lesions (median = 1 (0–56) than for the patients who did not (median = 3 (0–42)) (U = 3224, *p* = 0.002) (Table 3).

## 4. Discussion

The study found that 75.5% of the selected MS cases were CSF OCB positive. According to the literature, CSF OCBs are present in up to 95% of patients with multiple sclerosis; the positivity persists through the course of the disease and is considered to be the immunological hallmark of the disease [17]. A meta-analysis of 48 studies assessed the prevalence of OCBs in MS and showed that 87.7% of patients with MS were OCB positive [7]. Therefore, the OCB positivity in our study was even lower. It is worth mentioning that even though the role of OCBs in the global guidelines of MS diagnostics changed since the 2000s, it was routinely used in confirming the MS diagnosis in the LUHS Kaunas Clinics during the time period of our study (2000–2020). Additionally, although this long period includes different methods of the assessment of OCBs, in the Kaunas Clinics, CSF IgG OCBs were tested using isoelectric focusing and IgG specific immunofixation during those 20 years. In this study, as in many others, the prevalence of MS among women was higher (gender ratio 2.08). Studies in France, Canada and Denmark show similar but slightly higher results, where the gender ratio varies from 2.45 to 3.2 [18,19,20].

The mean age at the time of the symptom onset in the study was 32 years. This matches with other trials in Europe that show the mean age of symptom onset to be 31–33 years [18,21]. However, this study found no relation between the OCB positivity status and the age of symptom onset. The same results could be confirmed by the study performed in Turkey [22]. On the other hand, studies in Sweden and China have found that the age at the time of the first symptoms was earlier in OCB positive patients [23,24].

In this study, similar to the Swedish and Norwegian studies, OCB positive patients received their diagnosis earlier than OCB negative patients [23,25]. No statistically significant relation was found between OCB status and sex. Turkish, Norwegian and Chinese studies also reported the same result [22,24,25]. However, one Swedish study showed a higher prevalence of males in the OCB negative group [23]. An association with the course of the disease could not be analyzed, because only 10 out of 200 patients included in the study had PPMS or SPMS course. Other studies did not find a significant relation between OCB positivity status and the course of the disease [22,24], although some researchers report that OCB positivity is associated with primary progressive MS [26].

Other European studies found that OCB positive patients had a higher EDSS score, reached a higher EDSS score in a shorter period of time and had higher EDSS scores in younger ages [23,27], but in this study, there were no relations between OCB results and EDSS scores of the first and last visits, and its change through time. It could be hypothesized that a larger number of cases is needed to confirm the aforementioned results, or a relatively high percentage of OCB positive patients could distort the study results.

Based on the McDonald criteria, to confirm dissemination in space, typical lesions should be found in at least two of the following regions: juxtacortical, cortical, periventricular, infratentorial and spinal cord [5]. According to the MRI descriptions in the LUHS hospital, the CNS lesions were divided into the following six groups: periventricular, corpus callosum, brainstem, cerebellar, juxtacortical and spinal cord. By comparing the results of the first and the last MRI scan and assessing the appearance of new lesions, it was possible to determine whether the results of the MRI scan improved, stayed the same or worsened during the course of the disease. We also checked how the MRI results were affected with OCB positivity status, EDSS score and age at the time of the first symptoms and diagnosis.

The majority (81.3%) of patients who had lesions in the spinal cord were OCB positive, meaning that spinal cord lesions were more frequently found in OCB positive patients. This correlates with the results of a study that found an association between OCB positivity and the presence of lesions in the lower cervical spinal cord segments [22]. Other studies on MS lesions and OCBs found no significant relation between spinal cord lesions and OCB positivity [28,29]. Some studies even report that higher inflammatory activity in the spinal cord due to lesions could cause symptoms of a specific nature, for example, autonomic dysregulation [30,31]. Conflicting results from said studies show that further investigation is needed, and pathophysiological mechanisms of the association between OCB positivity and spinal cord lesions are still unclear. The fact that both OCB positivity [32] as well as spinal cord lesions [33] were linked with worse prognosis of the disease by other researchers could explain the association of these factors in our study. In this study, the spinal cord lesions were not divided into upper/lower spinal cord segments, and further investigation might be needed regarding this issue. Another limitation of our study is that the number of lesions in specific locations was not specified in the MRI descriptions; therefore, the feature is not included in this study. Additionally, only 141 out of 200 patients had MRI scans of the cervical spinal cord. Nonetheless, the lesions in the cervical spine are reported to cause disability more often, which confirms the idea of CSF OCBs to be a poor prognosis factor [34,35].

The study also showed that other lesion localizations (periventricular, c. callosum, brainstem, cerebellar, juxtacortical) in MRI were not associated with OCB positivity status. An association with the appearance of new lesions in MRI through the course of the disease and the number of MRI lesions was also not found. A study of Italian patients showed no link between lesion distribution and OCB positivity as well [36]. A Swedish study found that OCB positivity status was associated with a higher lesion load; however, the results were not statistically significant. Only a trend was seen for a more than two-fold risk of increased lesion burden in the infratentorial compartment in OCB positive patients [37]. A study which investigated the significance of OCB positivity and periventricular lesions found a possible association, but the course of the disease was not affected by the appearance of the lesions in this localization [38].

The study did not find a relation between either of the lesion localizations and patients’ age at the time of the first symptoms and diagnosis, but the patients with juxtacortical lesions were diagnosed faster than those without said lesions. D. Pareto et al. revealed that juxtacortical lesions are related to cortical thinning and subcortical gray matter volume loss [39]. These changes correlate with greater disability, cognitive impairment [40] and predict short-term physical worsening [41], which could explain why the aforementioned patients were diagnosed faster. Our study showed only a trend (*p* = 0.073) of patients with spinal cord lesions to have a greater EDSS score increase between their first and last visits. Similarly, a Dutch study revealed that the spinal cord (baseline) lesions were associated with a greater EDSS progression (in 6 and 11 years) compared to patients without said lesions. Dekker et al. also reported that while spinal cord lesions alone are linked with a worse progression of disability, the simultaneous presence of both spinal cord and infratentorial lesions are not. This means that different study designs might yield different results, as in our study, the lesion localizations were not grouped in any way. It also showed that lesions localized in the brainstem and in the cerebellum were not associated with a higher EDSS progression [33]. Our study did not associate cerebellar lesions and EDSS scores; however, we found that patients with lesions in the brainstem had a significantly higher EDSS score at the first visit, and the results correlate with the neuroanatomic importance of the brainstem. The score during the last visit was also higher in this patient group. Even so, the patients with brainstem lesions did not have a faster EDSS progression. Contrastingly, the lesions in the corpus callosum resulted in a greater EDSS increase, but not higher overall scores during the first and last visit. It might be hypothesized that the result could be due to the atrophy of corpus callosum, which is commonly found to correlate with disease progression [42].

The main advantage of this study is an extensive evaluation of various associations between OCB positivity, MRI data findings and MS features. This could lead to a further understanding of the disease, its progression and prognosis. Even though this study evaluated 200 MS cases, we believe that the main disadvantage of our study is the sample size, and further investigation with a wider selection of patients is necessary. Another limitation of our study is that the vast majority of the included cases had RRMS, meaning that the relations between the characteristics included in our study and the course of MS could not be analyzed with statistical significance. Additionally, to assess the course of the disease, we collected the EDSS scores and MRI data of the first and last visit, but the time between these visits was not included in this study. Another important issue is that in some cases, the MRI descriptions were not extensive, i.e., the number of lesions and exact lesion localizations were not specified. This additional information could allow a quantitative analysis of MRI changes. Additionally, even though one of the main results of our study is the significant relation between spinal MRI lesions and OCB positivity, not all patients included had MRI scans of the cervical spinal cord. Moreover, the data regarding the disease modifying treatment status was not gathered, even though it could have affected the results.

## 5. Conclusions

OCB positive patients were diagnosed with MS earlier and had spinal cord lesions more frequently than OCB negative patients. Patients with lesions in the corpus callosum had a greater increase in the EDSS score between their first and last visit. Patients with brainstem lesions had higher EDSS scores during their first and last visit. Even so, the progression of the EDSS score was not greater. The time between the onset of symptoms and the diagnosis was shorter for the patients who had juxtacortical lesions than for the patients who did not.

To provide more extensive and accurate results in further research, the sample size could be increased. The inclusion criteria could be modified, and only patients with cervical MRI should be included. The research design could also be improved by expanding the study by recording data about the type of first symptoms, disease modifying treatment and setting a follow-up period for a more accurate evaluation of the disease progression.

## Figures and Tables

**Table 1:** Baseline characteristics.

Characteristic	*n* (%)
Gender	
Male	65 (32.5)
Female	135 (67.5)
OCB status	
OCB positive	151 (75.5)
OCB negative	49 (24.5)
Course of the disease	
RR	190 (95)
PP	1 (0.5)
SP	9 (4.5)
Lesion localization	
Periventricular	184 (92)
C. callosum	148 (74)
Brainstem	100 (50)
Cerebellar	94 (47)
Juxtacortical	131 (66.2)
Spinal cord	80 (56.7)
Changes in MRI	
Negative	74 (37.4)
Stable/positive	124 (62.6)
**Characteristic**	**Mean (min/max)**
Age at first symptoms	32.13 ± 10.8 (12/73)
Age at diagnosis	36.4 ± 11.6 (18/74)
Time from first symptoms to diagnosis	5.07 ± 9.35 (0/56)
EDSS1	2.28 ± 1.16 (0/7)
EDSS2	3.17 ± 1.77 (0/9.5)
EDSS2–EDSS1	0.86 ± 1.66 (−4.5/6.5)

Abbreviations: OCBs—oligoclonal bands; MRI—magnetic resonance imaging; RR—relapsing–remitting multiple sclerosis; PP—primary progressive multiple sclerosis; SP—secondary progressive multiple sclerosis; EDSS1—Expanded Disability Status Scale score at the first visit; EDSS2—Expanded Disability Status Scale score at the last visit.

**Table 2:** Association of case characteristics and OCB status.

Characteristic		OCB+ *n*(%)	OCB− *n*(%)	*p* Value
Gender	Male	51 (78.5)	14 (21.5)	0.499
	Female	100 (74.1)	35 (25.9)	
Disease course	Relapsing–remitting	143 (75.3)	47 (24.7)	0.837
	Primary progressive	1 (100)	0 (0)	
	Secondary progressive	7 (77.8)	2 (22.2)	
MRI lesion localization	Periventricular lesions	141 (76.6)	43 (23.4)	0.207
	C. callosum	113 (76.4)	35 (23.6)	0.637
	Brainstem	81 (81)	19 (19)	0.071
	Cerebellar	74 (78.7)	20 (21.3)	0.318
	Juxtacortical	98 (74.8)	33 (25.2)	0.84
	Spinal cord	65 (81.3)	15 (18.7)	0.034
Changes in MRI (lesion count and size)	Negative	57 (77)	17 (23)	0.655
	Stable/positive	92 (74.2)	32 (25.8)	

Abbreviations: MRI—magnetic resonance imaging; OCB—oligoclonal band.

**Table 3:** Association of MRI lesion localizations and clinical features.

	Age at First Symptoms	Age at Diagnosis	Time from First Symptoms to Diagnosis	EDSS1	EDSS2	EDSS2-EDSS1
Periventricular	0.247	0.502	0.226	0.685	0.372	0.424
C. Callosum	0.505	0.338	0.366	0.371	0.085	0.045
Brainstem	0.184	0.117	0.824	0.002	0.004	0.128
Cerebellum	0.243	0.577	0.805	0.145	0.362	0.926
Juxtacortical	0.921	0.241	0.002	0.320	0.090	0.075
Spinal cord	0.479	0.496	0.555	0.339	0.143	0.073

Abbreviations: EDSS1—Expanded Disability Status Scale score at the first visit; EDSS2—Expanded Disability Status Scale score at the last visit.

## Data Availability

The research data are available upon request.

## References

[1] Walton C., King R., Rechtman L., Kaye W., Leray E., Marrie R.A., Robertson N., La Rocca N., Uitdehaag B., Van Der Mei I. (2020). Rising prevalence of multiple sclerosis worldwide: Insights from the Atlas of MS, third edition. Mult. Scler. J..

[2] Pellegrini F., Copetti M., Sormani M.P., Bovis F., de Moor C., Debray T.P., Kieseier B.C. (2019). Predicting disability progression in multiple sclerosis: Insights from advanced statistical modeling. Mult. Scler. J..

[3] Garg N., Smith T.W. (2015). An update on immunopathogenesis, diagnosis, and treatment of multiple sclerosis. Brain Behav..

[4] Rival M., Galoppin M., Thouvenot E. (2022). Biological Markers in Early Multiple Sclerosis: The Paved Way for Radiologically Isolated Syndrome. Front. Immunol..

[5] Thompson A.J., Banwell B.L., Barkhof F., Carroll W.M., Coetzee T., Comi G., Correale J., Fazekas F., Filippi M., Freedman M.S. (2018). Diagnosis of multiple sclerosis: 2017 revisions of the McDonald criteria. Lancet Neurol..

[6] Balnytė R., Matijošaitis V., Čelpačenko I., Malciūtė M., Stankevičiūtė R., Laucius O. (2022). Factors Related to the Progression of Clinically Isolated Syndrome to Multiple Sclerosis: A Retrospective Study in Lithuania. Medicina.

[7] Dobson R., Ramagopalan S., Davis A., Giovannoni G. (2013). Cerebrospinal fluid oligoclonal bands in multiple sclerosis and clinically isolated syndromes: A meta-analysis of prevalence, prognosis and effect of latitude. J. Neurol. Neurosurg. Psychiatry.

[8] Von Glehn F., Farias A.S., De Oliveira A.C.P., Damasceno A., Longhini A.L.F., Oliveira E.C., Damasceno B.P., Santos L.M.B., Brandão C.O. (2012). Disappearance of cerebrospinal fluid oligoclonal bands after natalizumab treatment of multiple sclerosis patients. Mult. Scler..

[9] Hemond C.C., Bakshi R. (2018). Magnetic resonance imaging in multiple sclerosis. Cold Spring Harb. Perspect. Med..

[10] Filippi M., Preziosa P., Banwell B.L., Barkhof F., Ciccarelli O., De Stefano N., Geurts J.J.G., Paul F., Reich D.S., Toosy A.T. (2019). Assessment of lesions on magnetic resonance imaging in multiple sclerosis: Practical guidelines. Brain.

[11] Sormani M.P., Bruzzi P. (2013). MRI lesions as a surrogate for relapses in multiple sclerosis: A meta-analysis of randomised trials. Lancet Neurol..

[12] Elliott C., Belachew S., Wolinsky J.S., Hauser S.L., Kappos L., Barkhof F., Bernasconi C., Fecker J., Model F., Wei W. (2019). Chronic white matter lesion activity predicts clinical progression in primary progressive multiple sclerosis. Brain.

[13] Ammitzbøll C., Dyrby T., Lyksborg M., Schreiber K., Ratzer R., Christensen J.R., Iversen P., Magyari M., Garde E., Sørensen P. (2018). Disability in progressive MS is associated with T2 lesion changes. Mult. Scler. Relat. Disord..

[14] Koch M.W., Mostert J., Repovic P., Bowen J.D., Strijbis E., Uitdehaag B., Cutter G. (2022). MRI brain volume loss, lesion burden, and clinical outcome in secondary progressive multiple sclerosis. Mult. Scler. J..

[15] Radue E.-W., Barkhof F., Kappos L., Sprenger T., Häring D.A., de Vera A., von Rosenstiel P., Bright J.R., Francis G., Cohen J.A. (2015). Correlation between brain volume loss and clinical and MRI outcomes in multiple sclerosis. Neurology.

[16] Charil A., Zijdenbos A.P., Taylor J., Boelman C., Worsley K.J., Evans A.C., Dagher A. (2003). Statistical mapping analysis of lesion location and neurological disability in multiple sclerosis: Application to 452 patient data sets. Neuroimage.

[17] Graner M., Pointon T., Manton S., Green M., Dennison K., Davis M., Braiotta G., Craft J., Edwards T., Polonsky B. (2020). Oligoclonal IgG antibodies in multiple sclerosis target patient-specific peptides. PLoS ONE.

[18] Leray E., Moreau T., Fromont A., Edan G. (2016). Epidemiology of multiple sclerosis. Rev. Neurol..

[19] Orton S.-M., Herrera B.M., Yee I.M., Valdar W., Ramagopalan S.V., Sadovnick A.D., Ebers G.C., Canadian Collaborative Study Group (2006). Sex ratio of multiple sclerosis in Canada: A longitudinal study. Lancet Neurol..

[20] Koch-Henriksen N., Sørensen P.S. (2010). The changing demographic pattern of multiple sclerosis epidemiology. Lancet Neurol..

[21] Wandall-Holm M.F., Andersen M.A., Buron M.D., Magyari M. (2022). Aging With Multiple Sclerosis: Age-Related Factors and Socioeconomic Risks. Front. Neurol..

[22] Kaya Tutar N., Söylemez E., Ömerhoca S., Kale İçen N. (2023). The Effect of Oligoclonal Bands in Patients with Multiple Sclerosis. Turk. J. Neurol..

[23] Karrenbauer V.D., Bedri S.K., Hillert J., Manouchehrinia A. (2021). Cerebrospinal fluid oligoclonal immunoglobulin gamma bands and long-term disability progression in multiple sclerosis: A retrospective cohort study. Sci. Rep..

[24] Lu T., Zhao L., Sun X., Au C., Huang Y., Yang Y., Bao J., Wu A., Kermode A.G., Qiu W. (2019). Comparison of multiple sclerosis patients with and without oligoclonal IgG bands in South China. J. Clin. Neurosci..

[25] Simonsen C.S., Flemmen H., Lauritzen T., Berg-Hansen P., Moen S.M., Celius E.G. (2020). The diagnostic value of IgG index versus oligoclonal bands in cerebrospinal fluid of patients with multiple sclerosis. Mult. Scler. J. Exp. Transl. Clin..

[26] Lourenco P., Shirani A., Saeedi J., Oger J., Schreiber W.E., Tremlett H. (2013). Oligoclonal bands and cerebrospinal fluid markers in multiple sclerosis: Associations with disease course and progression. Mult. Scler. J..

[27] Coll-Martinez C., Quintana E., Buxó M., Salavedra-Pont J., Gasull-Vicens L., Quiroga-Varela A., Costa-Frossard L., Villar L.M., Fernández-Díaz E., Gracia J. (2022). Oligoclonal IgM bands are a promising biomarker for long-term cognitive outcomes in multiple sclerosis. Mult. Scler. Relat. Disord..

[28] Ellidag H.Y., Eren E., Erdogan N., Ture S., Yilmaz N. (2013). Comparison of neurophysiological and mri findings of patients with multiple sclerosis using oligoclonal band technique. Ann. Neurosci..

[29] Zhao L., Abrigo J., Chen Q., Au C., Ng A., Fan P., Mok V., Qiu W., Kermode A.G., Lau A.Y. (2020). Advanced MRI features in relapsing multiple sclerosis patients with and without CSF oligoclonal IgG bands. Sci. Rep..

[30] Sirbu C.A., Mezei R.-M., Falup-Pecurariu C., Bratu O.G., Sirbu A.M., Ghinescu M.C., Radu F.I. (2020). Autonomic dysfunctions in multiple sclerosis: Challenges of clinical practice (Review). Exp. Ther. Med..

[31] Rzepiński Ł., Zawadka-Kunikowska M., Newton J.L., Zalewski P. (2021). Cardiac Autonomic Dysfunction in Myasthenia Gravis and Relapsing-Remitting Multiple Sclerosis—A Pilot Study. J. Clin. Med..

[32] Ben Noon G., Vigiser I., Shiner T., Kolb H., Karni A., Regev K. (2021). Reinforcing the evidence of oligoclonal bands as a prognostic factor in patients with Multiple sclerosis. Mult. Scler. Relat. Disord..

[33] Dekker I., Sombekke M.H., Balk L.J., Moraal B., Geurts J.J.G., Barkhof F., Uitdehaag B.M.J., Killestein J., Wattjes M.P. (2020). Infratentorial and spinal cord lesions: Cumulative predictors of long-term disability?. Mult Scler..

[34] Leguy S., Combès B., Bannier E., Kerbrat A. (2021). Prognostic value of spinal cord MRI in multiple sclerosis patients. Rev. Neurol..

[35] Kearney H., Miller D.H., Ciccarelli O. (2015). Spinal cord MRI in multiple sclerosis—Diagnostic, prognostic and clinical value. Nat. Rev. Neurol..

[36] Pichiecchio A., Tavazzi E., Maccabelli G., Ponzio M., Romani A., Schiappacassa R., Poloni G.U., Franciotta D., Roccatagliata L., Bergamaschi R. (2009). MR peri-CSF lesions and CSF oligoclonal bands in Italian multiple sclerosis patients. Acta Neurol. Scand..

[37] Karrenbauer V.D., Prejs R., Masterman T., Hillert J., Glaser A., Imrell K. (2013). Impact of cerebrospinal-fluid oligoclonal immunoglobulin bands and HLA-DRB1 risk alleles on brain magnetic-resonance-imaging lesion load in Swedish multiple sclerosis patients. J. Neuroimmunol..

[38] Akaishi T., Takahashi T., Nakashima I. (2018). Oligoclonal bands and periventricular lesions in multiple sclerosis will not increase blood-brain barrier permeability. J. Neurol. Sci..

[39] Pareto D., Sastre-Garriga J., Auger C., Vives-Gilabert Y., Delgado J., Tintoré M., Montalban X., Rovira A. (2015). Juxtacortical Lesions and Cortical Thinning in Multiple Sclerosis. Am. J. Neuroradiol..

[40] Bermel R., Bakshi R. (2006). The measurement and clinical relevance of brain atrophy in multiple sclerosis. Lancet Neurol..

[41] Gueye M., Cacciaguerra L., Tedone N., Vizzino C., Mistri D., Pagani E., Filippi M., Rocca M.A. (2022). Lesion Location Matters in Multiple Sclerosis: Clinical and Cognitive Correlates of Juxtacortical and Subventricular Zone Lesions (P1-1.Virtual). Neurology.

[42] Kale N., Agaoglu J., Tanik O. (2010). Electrophysiological and clinical correlates of corpus callosum atrophy in patients with multiple sclerosis. Neurol. Res..

